# Thyroid Hormone Supplementation Restores Cognitive Deficit, Insulin Signaling, and Neuroinflammation in the Hippocampus of a Sporadic Alzheimer’s-like Disease Rat Model

**DOI:** 10.3390/cells13211793

**Published:** 2024-10-30

**Authors:** Paulina Sepúlveda, Ana Flavia Fernandes Ferreira, Cristian Sandoval, Giovanna Bergoc, Ana Caroline Rippi Moreno, Maria Tereza Nunes, Andréa da Silva Torrão

**Affiliations:** 1 Departamento de Ciencias Preclínicas, Facultad de Medicina, Universidad de La Frontera, Temuco 4811230, Chile; 2 Departamento de Fisiologia e Biofísica, Instituto de Ciências Biomédicas, Universidade de São Paulo, São Paulo 05508-000, Brazil; anaffernandesf@gmail.com (A.F.F.F.); giovannabergoc@icb.usp.br (G.B.); arippimoreno@usp.br (A.C.R.M.); mtnunes@icb.usp.br (M.T.N.); 3 Escuela de Tecnología Médica, Facultad de Salud, Universidad Santo Tomás, Los Carreras 753, Osorno 5310431, Chile; cristian.sandoval@ufrontera.cl; 4 Departamento de Medicina Interna, Facultad de Medicina, Universidad de La Frontera, Temuco 4811230, Chile

**Keywords:** Alzheimer’s disease, insulin signaling, neurodegeneration, triiodothyronine, streptozotocin

## Abstract

Thyroid hormones play a crucial role in the development of the central nervous system and are considered pivotal to cognitive functions in the adult brain. Recently, thyroid dysfunction has been associated with Alzheimer’s disease. The aim of this study was to assess the neuroprotective effects of triiodothyronine (T3) on insulin signaling, neuroinflammation, apoptosis, and cognitive function in a streptozotocin (STZ)-induced sporadic Alzheimer’s disease-like model. Male Wistar rats underwent stereotaxic surgery for intracerebroventricular injections of streptozotocin (STZ; 2 mg/kg) or vehicle in the lateral ventricles to induce an AD-like model. The animals received a daily dose of 1.5 μg of T3/100 g body weight or the same volume of vehicle for 30 days and were subdivided into four experimental groups: (1) animals receiving citrate treated with saline (Control = CTL); (2) animals receiving citrate treated with T3 (T3); (3) animals receiving STZ treated with saline (STZ); and (4) animals receiving STZ treated with T3 (STZ + T3). The novel object recognition test was used to measure cognitive function. Serum analysis, real-time RT-PCR, immunohistochemistry, and immunoblotting analyses were also carried out. Our results demonstrated that T3 treatment reversed cognitive impairment and increased Akt and GSK3 phosphorylation in the treated group, while also reducing microglial activation (Iba-1) and GFAP expression (reactive astrocytes), along with TNF-α, IL-6, and IL-1β levels in the hippocampus. Additionally, T3 treatment increased levels of the anti-apoptotic protein Bcl-2 and reduced the expression of the pro-apoptotic protein BAX in the hippocampus. Our study demonstrated that T3 could potentially protect neurons in an AD model induced by STZ.

## 1. Introduction

Alzheimer’s disease (AD) is a chronic neurodegenerative disorder and the most prevalent form of dementia in elderly individuals [1]. Its incidence increases twofold roughly every 5 years beyond the age of 65 [2]. The main neuropathologic signs of AD are amyloid-β (Aβ) plaques and neurofibrillary tangles (NFTs), as well as activated microglia and astrocytes [3]. This makes neurons less functional and leads to cell death [4,5]. The aforementioned alterations are conspicuous in the cortical and hippocampal areas of the brain [6]. This leads to a decline in memory and cognitive function, accompanied by neurobehavioral abnormalities that significantly impact daily activities [7]. Therefore, it is crucial to investigate efficient therapeutic approaches to hindering the initiation and advancement of AD.

Both genetic and environmental factors contribute to the development of AD. In a small number of cases, genetics is what causes the familial form of AD (FAD). The sporadic form (sAD), on the other hand, is linked to environmental risk factors like heart disease, stroke, cancer, poor glucose tolerance, and diabetes mellitus (DM) [8]. Several studies suggest that the brain insulin signaling pathway is impaired in sAD pathology [9,10,11]. Researchers have found that the main reason why neurons die in sAD is a brain state called insulin-resistant brain state (IRBS) and a decrease in the brain’s ability to use glucose (cerebral glucose hypometabolism) [12,13]. This suggests that AD is actually a neuroendocrine disorder that resembles diabetes mellitus (DM) but at the cerebral level. It is denominated as type 3 diabetes by scientists [14]. At the molecular level, the trafficking of the amyloid precursor protein (APP) and the phosphorylation of tau protein via glycogen synthase kinase 3 (GSK3) activity are under the control of insulin signaling [15,16]. Aβ plaques and NFTs have significant impacts on neuronal degeneration, neuroinflammation, and oxidative stress [17,18,19].

Recently, thyroid dysregulation has been associated with AD [20,21,22]. There is growing evidence that, besides their role in glucose metabolism and insulin signaling in peripheral tissues, thyroid hormones (THs) can also regulate these parameters in the brain [23,24]. Thyroid gland disorders are one of the main causes of cognitive impairment [20,25]. It is known that the hippocampus is very sensitive to the actions of thyroid hormones due to the high expression of their receptors in this brain region, where T3 influences glial functions in the hippocampus [26]. In fact, giving rats with hypothyroidism T3 normalized the high levels of pro-inflammatory cytokines, which suggests that T3 has an anti-inflammatory effect [27]. Hypothyroidism is also linked to changes in the expression of important molecules for memory and synaptic plasticity, like protein kinase B (Akt) and GSK3 [28]. It has been postulated that THs could regulate the expression of the Brain-Derived Neurotrophic Factor (*BDNF*) gene and synaptic plasticity while also changing genes involved in brain cell death [29,30].

Streptozotocin (STZ), a glucosamine-nitrosourea compound, is widely used experimentally to produce a diabetes mellitus model in rodents. However, its intracerebroventricular (icv) administration has been employed as a representative model of sAD [31,32]. It provokes inflammation, disrupts brain insulin signaling, and promotes the accumulation of Aβ plaques, NFTs, and the induction of progressive cognitive impairment [33,34,35]. Therefore, the purpose of this study was to assess the neuroprotective effects of T3 on insulin signaling, neuroinflammation, apoptosis, and cognition function in an STZ-induced sAD-like model.

## 2. Materials and Methods

The animal experiments adhered to the ARRIVE criteria, the National Institutes of Health Guide for the Care and Use of Laboratory Animals (NIH Publications No. 8023, amended 1978), and the guidelines set by the National Council for the Control of Animal Experimentation (CONCEA, Brazil). The Ethical Committee for Animal Research of the Institute of Biomedical Sciences, University of São Paulo (CEUA), granted approval for all protocols (protocols No. 23/2016 and No. 4296070621). Experiments were carried out according to ethics guidelines to reduce quantity, as well as the distress experienced by animals.

### 2.1. Animals 

We included 52 male Wistar rats (12 weeks of age), with weights ranging from 300 to 350 g, obtained from the facility for Specific-Pathogen-Free (SPF) rat production at the Institute of Biomedical Sciences, University of São Paulo (Animal Facility Network at USP). The animals (groups of 3–4 per cage) were housed at 23 °C ± 2, under a 12 h light/12 h dark cycle (lights on at 06:00 h), and with food and water *ad libitum*. The animals were randomly divided into four experimental groups: (1) animals that were injected with citrate buffer intracerebroventricularly and were supplemented with saline for 30 days (CTL); (2) animals that were injected with STZ intracerebroventricularly and were supplemented with saline for 30 days (STZ); (3) animals that were injected with citrate buffer intracerebroventricularly and were supplemented with triiodothyronine (T3) for 30 days (T3); and (4) animals that were injected with STZ intracerebroventricularly and were supplemented with T3 for 30 days (STZ + T3). 

### 2.2. Procedures

#### 2.2.1. Neurosurgery and ICV-STZ Delivery 

To induce the AD model, stereotaxic neurosurgery was performed as previously described in the literature [36,37]. In summary, the animals were anesthetized with ketamine (90 mg/kg) and xylazine (8 mg/kg) and positioned in a stereotaxic apparatus (Kopf Instruments, Tujunga, CA, USA). Prior to surgery, the area of the incision was cleaned with alcohol and shaved. The rats were stabilized with ear bars on the stereotaxic device to keep lambda and bregma on the same plane. The injection of STZ was administered using a Hamilton^®^ syringe (Neuros Syringes-65460-02, Reno, NV, USA) following the coordinates relative to the bregma: AP − 0.8 mm; ML ± 1.4 mm; and DV − 3.4 mm [38]. The STZ (Sigma-Aldrich, St. Louis, MO, USA) was injected bilaterally into the lateral ventricles at a dosage of 2 mg/kg in 2 μL per side. In the CTL and T3 groups, an equivalent volume (2 μL per side) of citrate buffer (0.05 mol/L; pH 4.5) was injected. After the procedure, the animals received a polyantibiotic (0.1 mg i.m.; Laboratório Bravet, Rio de Janeiro, RJ, Brazil). Unfortunately, three animals did not survive the surgical procedure. The remaining animals were closely observed until complete recovery, and their overall health was monitored on a daily basis. 

#### 2.2.2. T3 Supplementation

**Figure 1:**
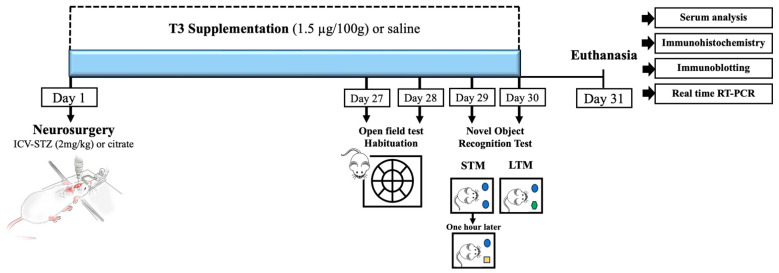
Graphical representation of the experimental design. The establishment of a streptozotocin (STZ)-induced sporadic Alzheimer’s disease (sAD) animal model, T3 supplementation, and experimental procedures are shown. STM—short-term memory; LTM—long-term memory.

The dosage of T3 in this experiment was determined based on studies conducted previously [28,39]. After neurosurgery, the animals received a daily dose of 1.5 μg/100 g body weight of triiodothyronine (T3, Sigma-Aldrich Co./T2877) or the equivalent volume of vehicle (saline solution) intraperitoneally (i.p.) for a period of 30 days, always at the same time. The initial administration for each animal was standardized at three minutes after surgery. A schematic representation of the experimental design and treatment timeline of the study is presented in Figure 1. 

#### 2.2.3. Novel Object Recognition Test (NOR) 

All behavioral tests were carried out between 7:00 a.m. and 1:00 p.m. at each time point following a 30 min acclimatization period for the animals in the testing room. In order to determine how the T3 supplementation affected the STZ-induced cognitive impairment, the animals (n = 12–13 per group) underwent the novel object recognition (NOR) test during the final week of supplementation (Figure 1). The novel object recognition test looks at non-spatial working memory, which is similar to episodic memory and depends on the cortex and hippocampus working properly [40,41]. The behavioral test consisted of three phases: the training phase, testing phase 1 (short-term memory), and testing phase 2 (long-term memory), as previously described [36,37]. In the training phase, rats were habituated to the open field arena (Ø = 60 cm; height = 50 cm) for 10 min, three times a day with intervals of 1 h, for 2 days, in the absence of any object. The first habituation session was used to evaluate the horizontal and vertical locomotion of the animal in the open field. Horizontal locomotion was quantified as the number of times that the animal changed quadrants on the ground, supporting all four paws, and vertical locomotion was quantified as the number of times that the animal adopted the posture of standing on its hind legs, with the trunk perpendicular to the ground.

During test phase 1, rats were exposed to two identical objects (A and A’) and were given 10 min to explore them. After 1 h, short-term memory (STM) was evaluated by presenting one of the training objects (A) and a different object (B) to the animals. Long-term memory (LTM) was tested after 24 h by presenting one of the training objects (A) and a new object (C) to the animals. Time spent in exploration was video-recorded, and exploration was defined as the time that the animal spent smelling or licking the object or touching it with its muzzle or front legs, or when the animal kept its muzzle at a distance of 1 cm or less from the object. These parameters were assessed for 5 min in each test. The discrimination index was established by dividing the time spent exploring the new object by the sum of the times spent exploring the new object and the familiar object.

#### 2.2.4. Serum Analysis

Blood was collected during sacrifice on day 31. The blood was centrifuged (3500 rpm for 15 min at room temperature), and the serum collected from the supernatant was used to evaluate the serum concentrations of thyroid-stimulating hormone (TSH), T3 (n = 5 per group), and BDNF (n = 6 per group). The concentrations were determined using specific kits following the manufacturer’s instructions for Luminex^®^ Xmap^®^ technology. For TSH and T3, the “MILLIPLEX Rat Thyroid Hormone Magnetic Bead Panel RTHYMAG-30k” kit (Merck, Darmstadt, Germany) was used, and for BDNF, the “Chemikine BDNF ELISA CYT 306” kit (ThermoFisher Scientific, Waltham, MA, USA) was employed [28,39].

#### 2.2.5. Real-Time RT-PCR

RNA extraction from hippocampal tissue samples (n = 5 per group) was isolated using 600 μL Trizol (Life Technologies, Carlsbad, CA, USA). To this, 120 µL of chloroform was added, followed by vortexing and centrifugation for 15 min at 12,000 rpm (Eppendorf centrifuge 5804R-rotor F45-30-11, Sigma-Aldrich Chemie GmbH, Schnelldorf, Germany) at 4 °C to separate phases. The upper phase was carefully transferred to a new microtube, to which 300 µL of chilled isopropanol was added. After mixing twice by inversion, the mixture rested at room temperature for 10 min and was then centrifuged for 10 min at 12,000 rpm at 4 °C. After discarding the supernatant, the RNA pellet was washed with 600 µL of 75% ethanol and centrifuged for 5 min at 7500 rpm at 4 °C, the supernatant was discarded, and the pellet was air-dried and stored at −80 °C. Subsequently, the pellet was resuspended in 30 µL of DEPC-treated water and heated at 65 °C for 5 min. RNA concentration (in µg/µL) was quantified using the Epoch Biotek equipment. For reverse transcription (RT), 1 µg of total RNA from each sample was used. The following components were added: 2 µL of Random Primer (Thermo Scientific No. 4368814), 10 mM of each dNTP, 2 µL of 5X Random Primer Buffer, 1 µL of enzyme (200 U/µL) Reverse Transcriptase, and DEPC-treated water to complete the solution up to 20 µL. The RT reaction was carried out at 25 °C for 10 min, followed by 37 °C for 120 min and 85 °C for 5 min. Real-time PCR used 1 µL of the RT product added to 10 µL of real-time PCR reaction mix containing 200 nM of each specific primer pair. Real-time PCR was performed using a Corbett instrument (Corbett Research, Sydney, Australia) under the following conditions: 50 °C for 2 min, 95 °C for 5 min, and 40 cycles of 95 °C for 20 s, 60 °C for 1 min, and 72 °C for 15 s. Results were analyzed using Rotor-Gene 6000 Series Software 1.7. Each sample reaction was performed in duplicate. The primer sequences (https://www.ncbi.nlm.nih.gov/gene/, accessed on 28 March 2021) were as follows 1. BDNF, Sense: AAGTGCCTTTGGAGCCTCCT; Anti-Sense: GCTAATACTGTCACACACGC—187 amplicon—NM_001270630.1, and 2. Rpl19, Sense: CCAATGAAACCAACGAAATCG; Anti-Sense: TCAGGCCATCTTTGATCAGCT—73 amplicon—NM_031103.1. The gene expression levels were calculated using the ΔΔCT method and normalized to Rpl19 as an internal control. Changes in gene expression among the groups were determined using the 2^−ΔΔCt^ method [42,43].

#### 2.2.6. Immunohistochemistry

Brain immunostaining was conducted as previously described [44]. Thirty-one days after stereotaxic surgery, four to five animals in each group were put to sleep with ketamine (100 mg/kg, i.p.) and xylazine (25 mg/kg, i.p.) and then underwent transcardiac perfusion with 0.9% saline solution and 4% paraformaldehyde solution (PFA). Brains were collected, fixed in 4% PFA for 4 h, and then kept in a 30% sucrose solution mixed with 0.1 M phosphate-buffered saline (PBS, pH 7.4). Tissues were cut on a sliding freezing microtome (Leica SM2000R Sliding Microtome, Leica^®^, Wetzlar, Germany) to a thickness of 30 µm. Slides containing the CA1, CA3, and dentate gyrus (DG) regions of the hippocampus were incubated overnight with primary antibodies at 1:1000; mouse monoclonal anti-glial fibrillary acidic protein (GFAP; Cat# G3893, Sigma-Aldrich Chemie GmbH, Schnelldorf, Germany), goat polyclonal anti-ionized calcium-binding adaptor molecule 1 (Iba1; Cat# ab5076, Abcam, Cambridge, MA, USA), and rabbit polyclonal anti-Interleukin-1 beta (IL-1β, Abcam Cat# ab9722). Subsequently, slides were washed and incubated for 2 h with biotinylated secondary antibodies at 1:200. Thereafter, an avidin–biotin complex (Cat# PK-7100, VECTASTAIN Elite ABC-Peroxidase Kit, Vector Laboratories, Newark, CA, USA) was added to the tissue, and it was marked with 0.05% 3–3′-diaminobenzidine (DAB) and 0.01% hydrogen peroxide in PBS. The tissue was then placed on glass coverslips with a subbing solution (0.05% chromium potassium sulfate and 0.5% gelatin). For the negative control, PBS without the primary antibody was used. This approach was chosen to make sure that any staining seen was not caused by non-specific antibody binding. 

#### 2.2.7. Image Acquisition and Quantification

Digital images were acquired using a Nikon E1000 microscope (Nikon Inc., Melville, NY, USA) and a Nikon DMX1200 digital camera (Nikon Imaging Software, v. 5.3, Nikon Inc., Melville, NY, USA) in fields located in sections 2.80 mm to 3.70 mm posterior to the Bregma. Images were captured using a 20× lens and analyzed with ImageJ software (version 1.54j, National Institutes of Health, Stapleton, NY, USA). The polygon tool was used to draw a selection around each area of interest in the CA1, CA3, and DG regions of the hippocampus, and the integrated optical density was measured. Five slices were analyzed. Ten replicates of each animal were analyzed, consisting of five randomly selected bilateral sections of each structure (the left and right hemispheres were analyzed). The means were normalized relative to the control. 

#### 2.2.8. Immunoblotting 

Quantification of protein levels was performed 31 days after stereotaxic surgery (n = 6–8 per group), following the methods previously described by our group [35,36,37]. The animals were decapitated, and their hippocampus were quickly removed. Hippocampus were dissected out and homogenized in the extraction buffer. The protein concentration of the samples was determined using the Bradford method (Bio-Rad, Hercules, CA, USA) and 30 μg of protein was loaded on standard SDS-PAGE gels (10% or 15% polyacrylamide minigels). A Bio-Rad semi-dry Trans-Blot cell system (Hercules, CA, USA) was then used to electrotransfer the proteins to nitrocellulose membranes. The membranes were then blocked for 2 h at room temperature with PBS containing 0.05% Tween-20 (TTBS) and 5% bovine serum albumin (BSA) and incubated overnight at 4 °C with the following specific primary antibodies: rabbit polyclonal anti-insulin receptor (IR, 1:1000; Cat# Sc-711, Santa Cruz Biotechnology, Dallas, TX, USA), rabbit monoclonal anti-Akt (1:2000; Cat# 4691, Cell Signalling Technology, Danvers, MA, USA), rabbit monoclonal anti-phospho-Akt (Ser473) (1:1000; Cat# 4060, Cell Signalling Technology, Danvers, MA, USA), mouse monoclonal anti-GSK3α/β (1:1000; Cat# Sc-56913, Santa Cruz Biotechnology, Dallas, TX, USA), rabbit polyclonal anti phospho-GSK3α/β (Ser21/9) (1:1000; Cat# 9331, Cell Signalling Technology, Danvers, MA, USA), rabbit monoclonal anti-Bcl2 (1:1000; Cat# 3498, Cell Signalling Technology, Danvers, MA, USA), rabbit Polyclonal anti Bax (1:1000; Cat# 2772, Cell Signalling Technology, Danvers, MA, USA), mouse monoclonal anti- GFAP (1:1000; Cat# G-3893, Sigma-Aldrich Chemie GmbH, Schnelldorf, Germany), rabbit Polyclonal anti-Tumor Necrosis Factor-α (TNF-α, 1:1000; Cat# ab6671, Abcam, Cambridge, MA, USA), mouse monoclonal anti-Interleukin-6 (IL-6, 1:500; Cat# ab9324, Abcam, Cambridge, MA, USA), and rabbit polyclonal anti-Monocarboxylate Transporter 8 (MCT8, 1:1000; Cat# ab192828, Abcam, Cambridge, MA, USA). In all of the experiments, a mouse monoclonal anti-Glyceraldehyde-3-Phosphate Dehydrogenase (GAPDH, 1:1000; Cat# 51332, Cell Signalling Technology, Danvers, MA, USA) was used as a loading control. Thereafter, membranes were incubated for 2 h with corresponding secondary antibodies. Blots were visualized using a chemiluminescence system in a digital scanner and analyzed for optical density in Image Studio software (LI-COR Image Studio Software, v. 6.0, LI-COR Biotechnology, NE, USA). Each experiment’s corresponding bands’ GAPDH-normalized percentages of protein change in relation to the control mean value.

#### 2.2.9. Statistical Analysis

The Kolmogorov–Smirnov test was utilized to verify the normal distribution prior to performing significance testing, and all of the data demonstrated adherence to the normal distribution. A two-way analysis of variance (ANOVA) test and a Tukey post hoc test were both used to compare the groups. The data were displayed as the average and standard deviations of the average. All statistical analyses were performed with a significance level of 5% (*p* ≤ 0.05). The data were analyzed in the statistical program GraphPad Prism^®^ software (Version 8, GraphPad Software Inc., San Diego, CA, USA). Upon reasonable request, the author will make the data available.

## 3. Results

### 3.1. Effects of T3 Supplementation on Thyroid Status in an STZ-Induced sAD-like Model

The thyroid status was assessed by detecting the serum levels of TSH and T3 after euthanasia (Table 1). No differences in total serum T3 concentration among groups were evident (*p* > 0.05). The levels of TSH in the blood dropped significantly in the groups that were given T3 compared to the groups that were not given the hormones (CLT vs. T3, *p* = 0.0001; and CTL vs. STZ + T3, *p* = 0.0001).

### 3.2. T3 Improves STZ-Induced sAD-like Cognitive Impairment

**Figure 2:**
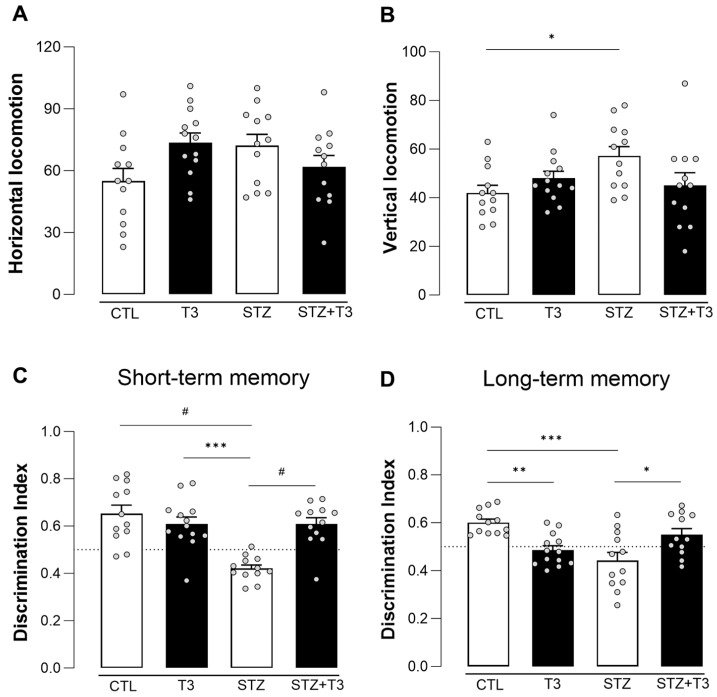
Results of open field and novel object recognition tests performed in animals after ICV injection of STZ and T3 supplementation. (**A**) Horizontal locomotion analysis in the open field test. (**B**) Vertical locomotion analysis in the open field test. (**C**) Discrimination index based on the object exploration time during the new object recognition memory test after one hour (short-term memory, STM) of the first exposure to objects. (**D**) Discrimination index based on the object exploration time during the new object recognition memory test after 24 h (long-term memory, LTM) of the first exposure to objects. The data are expressed as the mean ± SEM, determined through two-way analysis of variance (ANOVA), followed by Tukey’s post hoc test. (*) = *p* < 0.05; (**) = *p* < 0.01; (***) = *p* < 0.001; (#) = *p* < 0.0001, n = 12–13.

The NOR test was used between days 27 and 30 of the experiment to see how well the animals could tell the difference between familiar and unfamiliar objects (Figure 2). The goal of this experiment was to determine whether supplementation with T3 could reverse STZ-induced cognitive impairment. During the adaptation period of the NOR test, the open field test was conducted to rule out any deficits in motor activity among the animals. It is noteworthy that no significant differences were observed in horizontal locomotion between the groups. However, the vertical locomotion test revealed significant differences between the CTL and STZ groups (Figure 2B; *p* = 0.0410, F = 5.485). 

The NOR graphic displays a dashed line to represent equal exploration between the two objects, with a discrimination index of 0.5. The bars above the dashed line show that the animal exhibited a greater amount of exploration towards the new object. Our observations revealed that the STZ group experienced difficulty discriminating between objects in the short term (CTL vs. STZ; *p* = <0.0001, F = 17.29), and this impairment persisted even after 24 h (long-term memory) (CTL vs. STZ; *p* = <0.0002, F = 21.52). However, T3 supplementation reversed the cognitive impairment seen in both the short-term (STZ vs. STZ + T3; *p* = 0.0001, F = 17.29) and long-term (STZ vs. STZ + T3; *p* = 0.0144, F = 21.52) memory tests (Figure 2C,D, respectively). It is also observed that the T3 group shows a decrease in the discrimination index when compared to the control group in the long-term test (CTL vs. T3; *p* = 0.0075).

**Figure 3:**
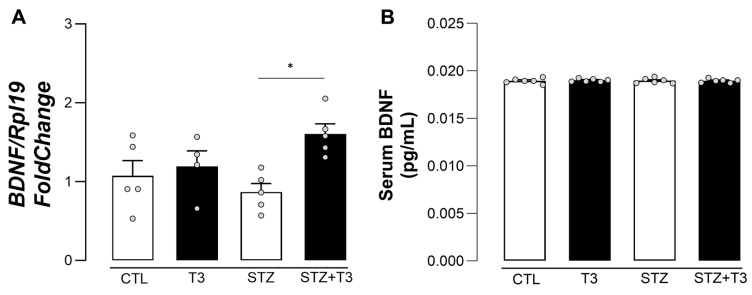
BDNF levels in animals after ICV injection of STZ and T3 supplementation. (**A**) Relative gene expression in the hippocampus. (**B**) Serum levels. The data are expressed as the mean ± SEM, determined through two-way analysis of variance (ANOVA), followed by Tukey’s post hoc test. (*) = *p* < 0.05, n = 5–6.

In addition to the behavioral changes, the gene expression levels of BDNF in the hippocampus of rats were analyzed. BDNF is highly expressed in the hippocampus and plays a crucial role in maintaining synaptic plasticity, thus aiding in the memory storage functions of the hippocampus [45]. In Figure 3A, it can be seen that the STZ group had less BDNF mRNA in their hippocampus than the STZ + T3 group (*p* = 0.0179, F = 3.841). Although variations were detected in BDNF mRNA (Figure 3A), no disparities were observed in BDNF serum levels among the different groups (Figure 3B).

### 3.3. Effects of T3 Supplementation on the Expression of MCT8 in an STZ-Induced sAD-like Model

**Figure 4:**
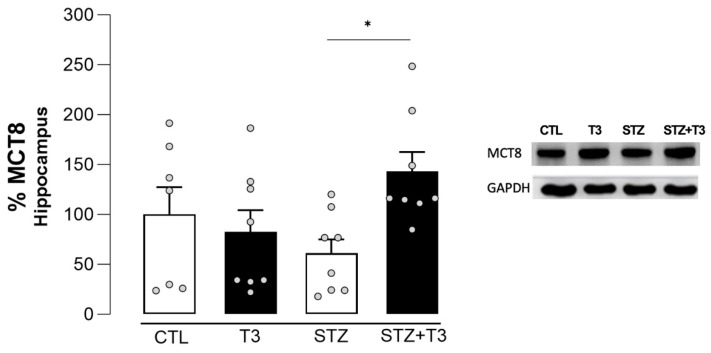
Quantification of the protein content detected by immunoblotting of the MCT8 receptor in the hippocampus of animals after ICV injection of STZ and supplementation with T3. The data are expressed as the mean ± SEM, determined through two-way analysis of variance (ANOVA), followed by Tukey’s post hoc test. (*) = *p* < 0.05, n = 7–8.

MCT8 is the specific transporter for thyroid hormones T4 and T3, allowing their entry into the brain through the blood–brain barrier [46]. We analyzed the expression of the TH transporter, MCT8, in the hippocampus (Figure 4). The results of our study indicate a significant difference between the STZ and STZ + T3 groups (*p* = 0.0396, F = 5.729). When compared to the STZ group, the STZ + T3 group shows a significant increase in the expression of the MCT8 transporter.

### 3.4. T3 Improves STZ-Induced sAD-like Alterations in Protein Expression Related to Insulin Signaling

**Figure 5:**
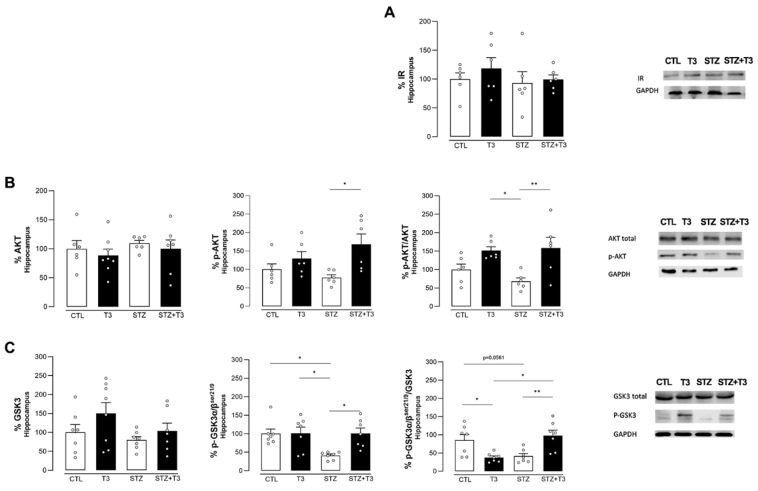
Quantification of proteins related to the insulin signaling pathway in the hippocampus of animals after ICV injection of STZ and supplementation with T3. (**A**) Total insulin receptor content. (**B**) Total AKT, p-AKT, and the ratio of p-AKT/total AKT. (**C**) GSK total, p-GSK3α/β, and the ratio of p-GSK3α/β to total GSK. The data are expressed as the mean ± SEM, determined through two-way analysis of variance (ANOVA), followed by Tukey’s post hoc test. (*) = *p* < 0.05; (**) = *p* < 0.01, n = 6–8.

T3 supplementation did not induce any alterations in the total quantity of insulin receptors (Figure 5A). Akt, a kinase protein, is involved in pro-survival pathways and becomes activated through phosphorylation (p-Akt). GSK-3β is a downstream target protein phosphorylated by p-Akt, leading to its inactivation. In its active form, GSK-3β regulates several pro-apoptotic pathways [47]. A rise in Akt phosphorylation, shown by the ratio of p-Akt/Akt (Figure 5B), suggests that this protein was activated more in the STZ + T3 group compared to the STZ group (*p* = 0.0142, F = 0.0084).

The amount of phosphorylated GSK-3α/β at serine 21/9 in the hippocampus was lower in the STZ group compared to the control (*p* = 0.0117, F = 5.191). This decrease was regulated through T3 supplementation, resulting in a higher level of phosphorylated GSK3α/β in the STZ + T3 group compared to the STZ group (STZ vs. STZ + T3; *p* = 0.0155) (Figure 5C). When GSK3α/β is phosphorylated at serine 21/9, this process has an inhibitory effect on the protein. Similarly, the p-GSK3 α/β/GSK ratios showed an increase in the STZ + T3 group compared to the STZ group (*p* = 0.0095, F = 20.90).

### 3.5. T3 Regulates Protein Expression Associated with Apoptotic Pathways in STZ-Induced sAD-like Model

**Figure 6:**
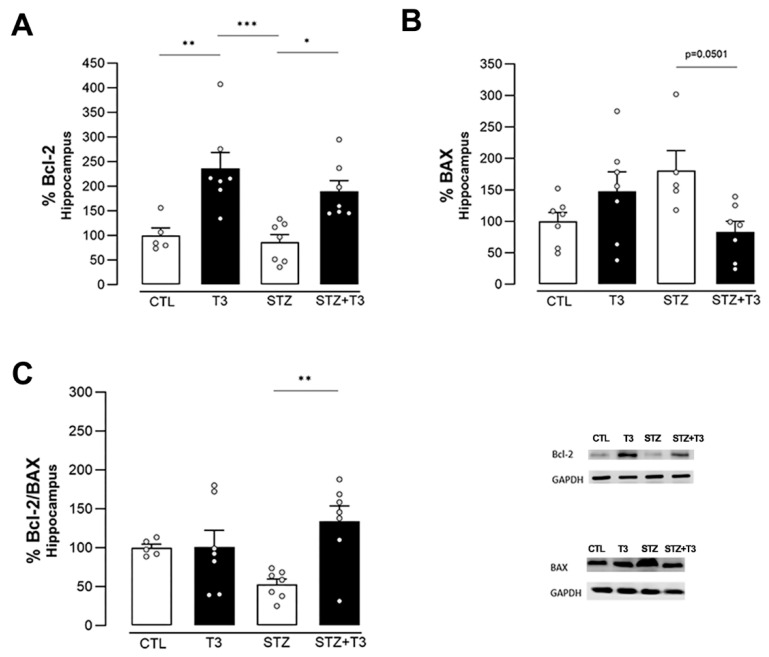
Quantification of proteins associated with apoptotic pathways in the hippocampus of animals after ICV injection of STZ and supplementation with T3. (**A**) Bcl-2. (**B**) BAX. (**C**) Ratio of Bcl-2/BAX. The data are expressed as the mean ± SEM, determined through two-way analysis of variance (ANOVA), followed by Tukey’s post hoc test. (*) = *p* < 0.05; (**) = *p* < 0.01; (***) = *p* < 0.001, n = 5–8.

One measure of apoptotic activity was the ratio of the antiapoptotic protein Bcl-2 to the pro-apoptotic protein BAX (Figure 6C). This was assessed to see how T3 affected the pathways that control apoptosis. It was observed that the expression of the Bcl-2 protein (CTL vs. T3; *p* = 0.0042, F = 0.4756; and STZ vs. STZ + T3, *p* = 0.0190) was higher in the T3-treated groups, which could suggest a neuroprotective effect of T3 (Figure 6A). When looking at the expression of the BAX protein, the STZ + T3 group had less protein than the STZ group, but the difference was not statistically significant (*p* = 0.0501) (Figure 6B). As shown in Figure 5C, however, the antiapoptotic stimulus was stronger in the STZ + T3 group compared to the STZ group (*p* = 0.0067, F = 6.024).

### 3.6. T3 Reduced Glial Cell Activation and Inflammatory Expression in the Hippocampus in STZ-Induced sAD-like Model

**Figure 7:**
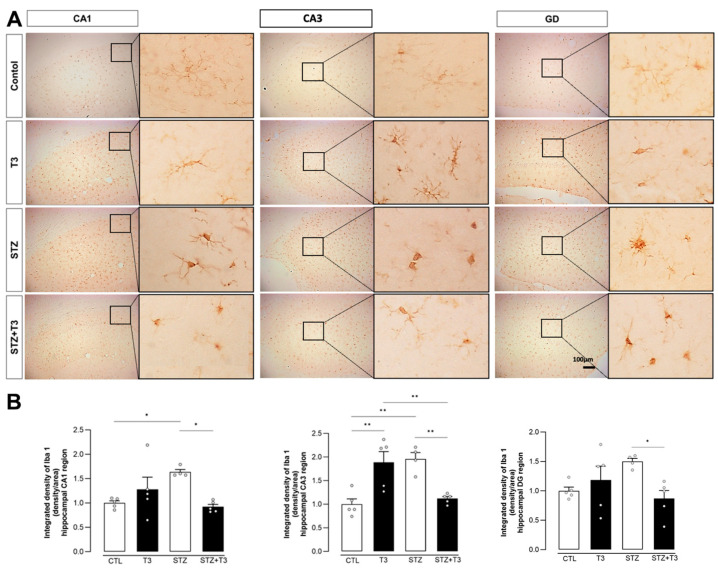
Immunohistochemistry and integrated density of Iba-1 in the hippocampal CA1, CA3, and DG regions of animals after ICV injection of STZ and supplementation with T3. (**A**) Digital images of coronal sections of the hippocampus show a reduction in Iba-1 immunostaining of STZ + T3 group when compared to STZ group. (**B**) Semi-quantitative analysis of Iba-1 immunostaining. The data are expressed as the mean ± SEM, determined through two-way analysis of variance (ANOVA), followed by Tukey’s post hoc test. (*) = *p* < 0.05; (**) = *p* < 0.01, n = 4–5.

Neuroinflammation, arising from the activation of glial cells and the release of various cytokines, plays a crucial role in the neuropathological alterations seen in Alzheimer’s disease [19]. Iba-1 is a microglial and macrophage-specific protein reported to be upregulated in activated microglia [48]. To assess the effects of T3 on microglial activation, we stained the CA1, CA3, and DG regions of the hippocampus with Iba-1. We observed an increase in Iba-1 staining in the CA1 and CA3 analyzed regions in the STZ groups compared to the respective CA1 and CA3 control groups (*p* = 0.0330 and *p* = 0.0031, respectively). Our results show that microglial activation is reduced in CA1, CA3, and DG after T3 supplementation (Figure 7) in the STZ + T3 group compared to the STZ group (STZ vs. STZ + T3, CA1 *p* = 0.0158; CA3 *p* = 0.0076; DG *p* = 0.0489).

**Figure 8:**
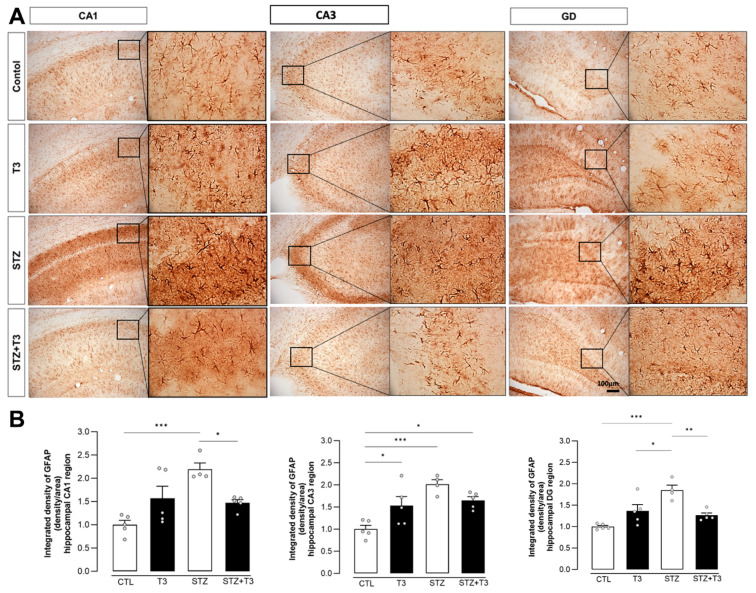
Immunohistochemistry and integrated density of GFAP in the hippocampal CA1, CA3, and DG regions of animals after ICV injection of STZ and supplementation with T3. (**A**) Digital images of coronal sections of the hippocampus show a reduction in GFAP immunostaining in STZ + T3 group compared to STZ group. (**B**) Semi-quantitative analysis of GFAP immunostaining. The data are expressed as the mean ± SEM, determined through two-way analysis of variance (ANOVA), followed by Tukey’s post hoc test. (*) = *p* < 0.05; (**) = *p* < 0.01; (***) = *p* < 0.001, n = 4–5.

GFAP is a protein that is linked to reactive astrocytes [49]. To see how T3 affected astrocyte reactivity, we used GFAP to stain the CA1, CA3, and DG regions of the hippocampus. In CA1, CA2, and DG, the upregulation of GFAP was also evident in the STZ group compared to the CTL group (*p* = 0.0007, *p* = 0.0005, and *p* = 0.0001, respectively). In addition, we found that GFAP expression was also reduced by T3 supplementation in the STZ + T3 group compared to the STZ group in the CA1 and DG regions of the hippocampus (*p* = 0.0354 and *p* = 0.0039, respectively) (Figure 8).

**Figure 9:**
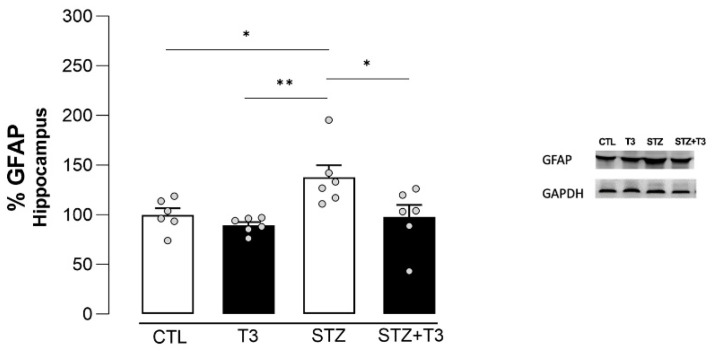
Quantification of the protein content detected by the immunoblotting of the GFAP in the hippocampus of animals after ICV injection of STZ and supplementation with T3. The data are expressed as the mean ± SEM, determined through two-way analysis of variance (ANOVA), followed by Tukey’s post hoc test. (*) = *p* < 0.05; (**) = *p* < 0.01, n = 6.

To complement this analysis, the protein expression of GFAP for immunoblotting in the hippocampus was also evaluated. It was observed that the STZ animals exhibited an increase in the expression of this protein compared to the CTL group (*p* = 0.0475; F = 0.0326). However, this increase was reversed by supplementation with T3 in the STZ + T3 group in the hippocampus (*p* = 0.0326) (Figure 9).

**Figure 10:**
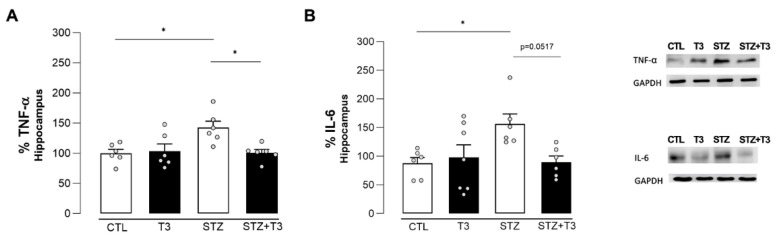
Quantification of cytokines in the hippocampus of animals after ICV injection of STZ and supplementation with T3. (**A**) TNF-α. (**B**) IL-6. The data are expressed as the mean ± SEM determined through two-way analysis of variance (ANOVA), followed by Tukey’s post hoc test. (*) = *p* < 0.05, n = 6–7.

The protein expression of IL-6 and TNF-α was also analyzed. In Alzheimer’s disease, there are many inflammatory cytokines near Aβ plaques and neurofibrillary tangles composed of TAU protein [50]. An important difference was seen between the STZ group and the CTL group in the amount of TNF-α protein expressed in the hippocampus (*p* = 0.015, F = 6.467). T3 supplementation was able to reduce this increase in the STZ + T3 group compared to the STZ group (*p* = 0.0174) (Figure 10). In the same way, IL-6 also showed a significant increase in the STZ group compared to the CTL group (*p* = 0.0422, F = 5.316), and the STZ + T3 group demonstrated a decrease in protein content compared to the STZ group, although not statistically significant (*p* = 0.0517).

**Figure 11:**
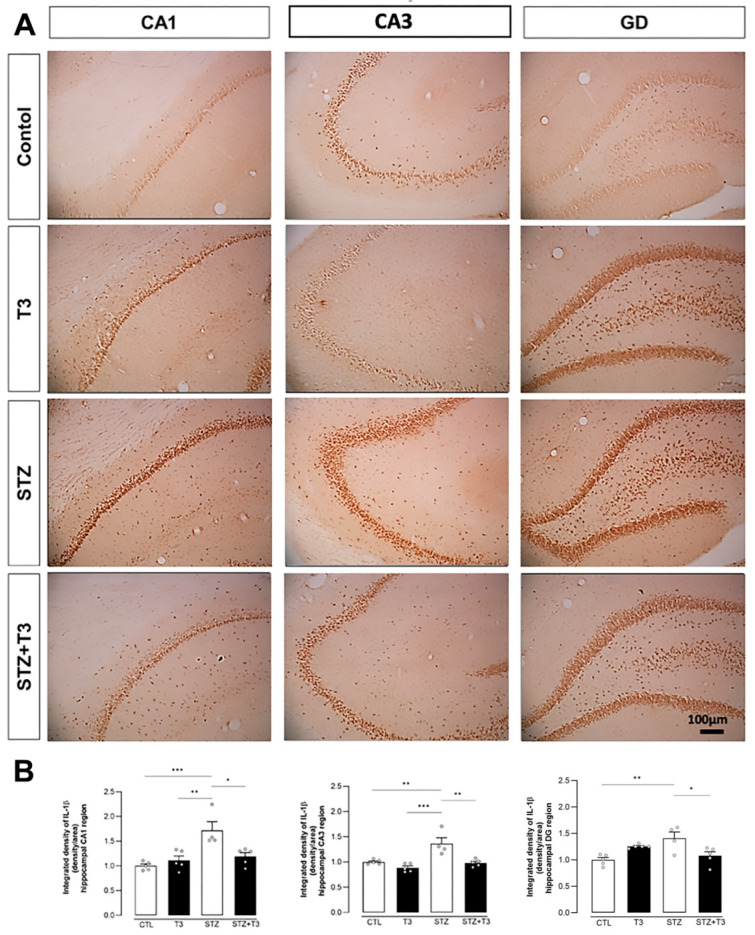
Immunohistochemistry and integrated density of IL-1β in the hippocampal CA1, CA3, and DG regions of animals after ICV injection of STZ and supplementation with T3. (**A**) Digital images of coronal sections of the hippocampus shows a reduction in IL-1β immunostaining in STZ + T3 group compared to STZ group. (**B**) Semi-quantitative analysis of IL-1β immunostaining. The data are expressed as the mean ± SEM, determined through two-way analysis of variance (ANOVA), followed by Tukey’s post hoc (*) = *p* < 0.05; (**) = *p* < 0.01; (***) = *p* < 0.001, n = 4–5.

Finally, immunohistochemistry was performed in the CA1, CA3, and DG regions to evaluate the cytokine IL-1β expression. This cytokine’s expression was found to be significantly higher in the STZ groups compared to the CTL groups in all three areas (*p* = 0.0008, *p* = 0.0027, and *p* = 0.0047, respectively). It was also noted that T3 supplementation significantly decreased the expression in the STZ + T3 group compared to the STZ group (*p* = 0.0105, *p* = 0.0018, and *p* = 0.0226, respectively) (Figure 11).

## 4. Discussion

This study investigated the effects of T3 supplementation for 30 days on cognition, insulin signaling, neuroinflammation, and apoptosis in a sAD experimental model. We showed that chronic T3 supplementation reverses cognitive impairment, increases the mRNA expression of BDNF, modifies some protein alterations in the insulin signaling pathway, regulates apoptotic pathways, and reduces glial cell activation and inflammatory expression in the hippocampus of animals after ICV injection of STZ. So, our study showed that T3 might help protect neurons in a model of sAD that was caused by STZ.

In the context of Alzheimer’s disease, the administration of low doses of STZ through ICV injection has been linked to changes in morphology, molecular processes, and behavior that resemble those observed in sporadic Alzheimer’s disease [35,37]. Our research team has shown that injecting 3 mg/kg of STZ into the brain’s ventricles causes cognitive decline within three hours, lasting for one month [35]. Our results showed that giving the animals in the STZ group a lower dose of STZ (2 mg/kg) still caused memory loss, apoptotic cell death, changes in insulin signaling, and inflammation (Figure 2).

The plasma levels of TSH observed in our study (Table 1) confirmed the effectiveness of our supplementation and are consistent with previous studies [28,39]. In hyperthyroidism, the interaction of T3 in the pituitary inhibits the expression of the gene encoding TSH and its secretion. Our results showed that animals receiving T3 supplementation (T3 and STZ + T3 groups) had lower levels of TSH, and T3 concentrations were maintained. Thyroid hormones are the main regulators of TSH secretion in a classic negative feedback system. T3 suppresses TRH, leading to the suppression of TSH production and secretion through the hypothalamus [51].

Regarding the behavioral analyses of animals’ locomotor activity in the open field, the groups did not show differences in horizontal locomotion rates (quadrant changes). With this result, we ruled out potential motor impairments that could influence their behaviors in the object recognition test [36,37]. T3 administration for 30 days reversed STZ-induced memory impairment in both short- and long-term object recognition tests, consistent with prior research highlighting thyroid hormones’ positive impact on cognition and memory [27,52]. Studies indicate that T3 supplementation in rats with induced hypothyroidism improves learning delays and memory deficits in the Morris water maze [27,28,29,30,31,32,33,34,35,36,37,38,39,40,41,42,43,44,45,46,47,48,49,50,51,52,53]. Furthermore, thyroid hormones enhance spike amplitudes and action potential frequencies in cultured hippocampal neurons in neonatal rats [54] and regulate neuronal excitability [55]. T3 has also been shown to reduce neural damage, enhance memory, and increase neuronal activation in the dentate gyrus of Alzheimer’s disease model animals [56]. Interestingly, in the long-term test, the T3 group exhibited memory impairment, suggesting a potential negative effect of elevated T3 levels in normal rats, consistent with the literature indicating that both high and low T3 levels in normal individuals can lead to cognitive impairments and behavioral changes [57,58].

BDNF is a significant neurotrophin involved in neurogenesis, neurotransmitter synthesis, synaptic plasticity, and neuronal protection. STZ injection resulted in a reduction in BDNF gene expression in the hippocampus, as previously shown [59]. BDNF concentrations in the brain and blood are involved in the development of Alzheimer’s disease [60]. During our study, the amount of BDNF in the blood did not change (Figure 3). However, the STZ + T3 group exhibited an increase in BDNF gene expression in the hippocampus, which may have helped protect neurons and changed how glial cells were activated. Research has shown that T3 controls the expression of the BDNF gene in several areas of the brain [29,61]. In addition, the fact that the STZ group’s cognitive impairment improved after T3 supplementation could mean that BDNF had a neurotrophic effect in the hippocampus [61,62]. The increase in BDNF mRNA levels in the T3-treated group suggests a direct modulation of brain gene expression (Figure 3A). Despite its crucial role in neuronal function and plasticity, the lack of changes in serum BDNF levels raises questions about the physiological relevance of these findings. Further research may be needed to better understand the relationship between changes in BDNF mRNA levels in the brain and serum levels, as well as their impact on cognitive function and the progression of Alzheimer’s disease [60].

Deficiencies or mutations in the MCT8 transporter, which allows the entry of THs through the blood–brain barrier, are characterized by cognitive and motor deficits resulting from cerebral hypometabolism [63,64]. Our results suggest a positive effect from the supplementation, since the STZ + T3 group showed an increase in the expression of this transporter in the hippocampus (Figure 4). Patients with AD show a decrease in cerebral blood flow [65], resulting in a reduced arrival of T3 to the brain. Moreover, thyroid hormones are associated with alterations in cerebral blood flow, as hypothyroid patients exhibit a global decrease in cerebral perfusion [65]. This could indicate that T3 supplementation improved blood flow and its arrival into the brain, leading to greater availability of T3 at the blood–brain barrier. MCT8 is the specific transporter for thyroid hormones, and its increased expression facilitates the passage of hormones to the brain [66,67].

In terms of cerebral insulin signaling, although we observed no differences in the total amount of IR protein between the groups (Figure 5A), there was an increase in Akt phosphorylation in the hippocampus of STZ animals supplemented with T3 (Figure 5B). This suggests a potential improvement in cerebral insulin signaling. Given that Akt is a crucial protein for cell proliferation, differentiation, and survival [68], this implies that T3 might have positively influenced the activation of the insulin signaling pathway, altered by the injection of STZ. It has been demonstrated that T3 rapidly activates the PI3K/AKT pathway in insulin signaling in the brain [57]. In fact, a previous study of our group showed that chronic administration of T3 (1.5 μg/100 g body weight) effectively reversed alterations in insulin signaling in the brains of diabetic rats by enhancing AKT phosphorylation and, consequently, decreasing GSK3β activation [28].

It is also interesting that we have seen that T3 can change the GSK3 protein phosphorylation compromised by STZ in the hippocampus by activating AKT (Figure 5C). AKT activation inhibited GSK3 by phosphorylation at Ser9 in GSK3β and Ser21 in GSK3α. Therefore, the inactivation of GSK3-β may be an important neuroprotective factor in Alzheimer’s disease, since activated GSK3 affects β-amyloid metabolism and tau protein phosphorylation. Moreover, the literature suggests that the GSK3 protein is a possible key mediator of apoptosis, contributing directly to neuronal loss [15,47]. Thus, T3 normalized the expression of proteins in the insulin signaling pathway in the hippocampus, emphasizing the potential neuroprotective role of T3 in Alzheimer’s disease, which aligns with the literature [23,69].

GSK3 exhibits pro-apoptotic functions when the PI3K-AKT pathway is inactive, as it regulates proteins of the BCL-2 family [47]. Our study further explored a potential signaling pathway that could mediate T3 neuroprotection through the AKT/GSK3/Bcl-2 pathway (Figure 5B,C and Figure 6A). This involves activating the signaling pathway and increasing the Bcl-2/Bax ratio in the hippocampus (Figure 6C). Our results revealed that the ratios of p-AKT/AKT, p-GSK-3α/β/GSK3, and Bcl-2/BAX were restored after T3 supplementation.

The Bcl-2 and BAX proteins, pivotal players in apoptotic cell death, exhibit opposing functions. While Bcl-2 fosters cell survival, BAX promotes cell death. Hypothyroidism has been demonstrated to modify the expression of genes in the BCL-2 family, inducing apoptosis in the brain by diminishing the levels of the Bcl-2 protein, a cell death preventer, and escalating the levels of the BAX protein, which triggers cell death [30]. T3 supplementation could ameliorate the apoptotic stimulus, either by elevating the expression of the Bcl-2 protein (Figure 6A) or reducing the expression of the BAX protein in the hippocampus (Figure 6B). Both T3 and T4 are recognized not only for diminishing certain pro-apoptotic markers, such as Caspase-3 and BAX, but also for their anti-apoptotic function by augmenting Bcl-2 [70]. Low levels of Bcl-2 have been associated with neuronal apoptosis induced by Aβ [68]. Elevating these levels has decelerated Tau hyperphosphorylation and diminished Aβ deposits outside of cells [71]. Our findings indicate that STZ modified the Bcl-2/BAX ratio in the hippocampus (Figure 6C).

STZ-induced neuroinflammation is a crucial component of our research, playing a fundamental role in neurodegenerative diseases [19]. This phenomenon is characterized by an increase in activated glial cells and proinflammatory cytokines [17,34]. The link between inflammation and cognitive impairment induced by STZ has been widely recognized [31,32]. We assessed both pro-inflammatory cytokines (IL-6, TNFα, and IL-1β) and glial markers (Iba-1 and GFAP) in the hippocampus (Figure 7, Figure 8 and Figure 9). Animals in the STZ group showed elevated protein expression of GFAP, IL6, and TNFα, along with an increase in immunoreactivity for GFAP, Iba-1, and IL-1β in the evaluated regions (CA1, CA3, and DG; Figure 7, Figure 9, and Figure 11). Glial cells, particularly astrocytes and microglia, are considered key regulatory elements in the functional plasticity of neural networks, and their activation promotes neurodegeneration and cognitive impairment [18,19,50].

Higher levels of pro-inflammatory cytokines in the brain are linked to hippocampus-dependent memory deficit and worsen inflammation in Alzheimer’s disease [17,61,72]. These changes in microglia occur in Alzheimer’s disease and result in synaptic dysfunction, neuronal death, and the inhibition of neurogenesis [73], which can accelerate the progression of the disease [50]. Additionally, astrocytes also play a neuroprotective role in Alzheimer’s disease by reducing plaque accumulation through Aβ clearance. Inflammatory cytokines such as tumor necrosis factor-alpha (TNF-α) and interleukin-1beta (IL-1β) can further activate glial cells, affecting cognitive and memory functions (3). Our T3 supplementation brought the levels of pro-inflammatory cytokines and glial activity markers in the hippocampus back to normal, consequently improving cognition in STZ animals supplemented with T3 (Figure 10 and Figure 11). The literature also indicates that T3 is a signaling factor that affects glial functions in the hippocampus. For instance, the administration of T3 normalized the increased expression of the pro-inflammatory cytokines IL-1β, IL-6, and TNF-α in the hippocampus of rats with hypothyroidism [27].

### Scope and Limitations

Our study shows that T3 can control the Bcl-2/BAX apoptotic pathway through PI3K/AKT activation in the brain. No other research has talked about this process in nervous tissue. However, future studies should also evaluate the expression of caspases. Another limitation of the study is that only male rats were used, which limits the generalizability of the results to both male and female human populations. It would be beneficial to consider future research that includes both males and females to achieve a more comprehensive understanding of the biological and pathological responses investigated.

## 5. Conclusions

Our findings suggest that THs could serve as potential adjuvant therapeutic candidates for the treatment of sporadic Alzheimer’s disease. Further studies are needed to delve into and comprehend the mechanisms of action of THs in the adult brain and how alterations in these hormones may impact the onset or progression of Alzheimer’s disease. The data that we have collected open up possibilities for the development of new alternative therapeutic approaches that could be applied to patients with Alzheimer’s disease.

## Figures and Tables

**Table 1:** Serum levels of TSH and T3 hormones in animals after ICV injection of STZ and T3 supplementation.

Parameters	Mean ± SEM				N per Group
	CTL	T3	STZ	STZ + T3	
T3 (pg mL^−1^)	3142.80 ± 163.20	3242.60 ± 168.50	3494.00 ± 282.70	3493.20 ± 331.70	5
TSH (pg mL^−1^)	2499.59 ± 1231.86	61.81 ± 13.24 ^a^	2401.37 ± 897.15	75.32 ± 36.05 ^a^	5

Data are expressed as the mean ± SEM, determined through two-way analysis of variance (ANOVA), followed by Tukey’s post hoc test. ^a^ Significant differences (*p* < 0.001) with the CTL group.

## Data Availability

The original contributions presented in the study are available on request.
